# Energy-efficient fragmentation-aware dual adaptive collision-free bit mapping MAC protocol for VANET

**DOI:** 10.1038/s41598-026-44566-6

**Published:** 2026-04-04

**Authors:** Fuhid Alanazi, Manoj Tolani, Sultan Alanazi, Arpita Kadel, Faisal Mohammed Alotaibi, Nasser S. Albalawi

**Affiliations:** 1 Faculty of Computer and Information Systems, Islamic University of Madinah, Madinah, 42351 Saudi Arabia; 2 Department of Electronics and Communication Technology, Jaypee Institute of Information Technology, Noida, 201309 Uttar Pradesh India; 3 Department of Computer Science, College of Computer Engineering and Science, Prince Sattam bin Abdulaziz University, Al-Kharj, 16273 Saudi Arabia; 4 Department of Electronics and Instrumentation Engineering, Shri G S Institute of Technology and Science, Indore, 452003 Madhya Pradesh India; 5 Department of Information System, College of Computer Engineering and Science, Prince Sattam bin Abdulaziz University, Al-Kharj, 16273 Saudi Arabia; 6 Department of Computer Sciences, Faculty of Computing and Information Technology, Northern Border University, Rafha, 91911 Saudi Arabia

**Keywords:** Medium access control, Fragmentation, Bit-mapping, Vehicular ad-hoc network, Energy science and technology, Engineering, Mathematics and computing

## Abstract

In the present work, an enhanced energy-efficient medium access control (MAC) protocol is proposed for adaptive data traffic conditions in VANET. The earlier adaptive collision-free MAC-bit map assisted (ACFM-BMA) protocol effectively integrated electric vehicle (EV) battery levels and buffer status to optimize communication efficiency and energy conservation. However, a key limitation was the underutilization of allocated slots by certain devices. To address this, we introduce the adaptive bit-map assisted protocol (ACFM-ABMA MAC), where each slot is dynamically fragmented into four subslots using two additional control bits, allowing vehicles to request only the required portion of a slot (e.g., 00 for 25% up to 11 for 100%). This fine-grained allocation reduces slot wastage, ensures better channel utilization, and further enhances energy savings. The proposed protocol is analyzed under low, moderate, and high data traffic scenarios, considering vehicle density, periodic traffic generation, and event-driven traffic probabilities. Simulation results confirm that ACFM-ABMA MAC achieves superior energy efficiency, and bounded latency across low, moderate, and high event-driven traffic scenarios.

## Introduction

Vehicular Ad Hoc Networks (VANETs) have emerged as a pivotal component of Intelligent Transportation Systems (ITS), enabling real-time communication among vehicles and between vehicles and roadside infrastructure to enhance road safety, traffic efficiency, and overall driving comfort. With the growing adoption of electric vehicles (EVs) and the integration of autonomous driving technologies, the role of communication protocols has become increasingly significant for both safety-critical and infotainment applications. Traditional VANET architectures based on IEEE 802.11p, also referred to as Dedicated Short-Range Communication (DSRC), employ contention-based mechanisms such as Carrier Sense Multiple Access with Collision Avoidance (CSMA/CA) for medium access. While these protocols offer simplicity, they suffer from unpredictable delays, higher collision probability in dense networks, and poor scalability, thereby limiting their suitability for time-sensitive vehicular communications^1,2^. To address these challenges, research has shifted toward contention-free and hybrid MAC designs that incorporate coordination, scheduling, and energy-aware features^3,4^.

Energy efficiency remains a crucial requirement in VANETs, particularly due to the reliance on battery-powered subsystems in EVs. Communication modules, sensors, and onboard processing units consume a significant share of the vehicle’s energy budget, which can reduce operational range and impair emergency communication when battery levels are critically low^5^. Furthermore, inefficient medium utilization in MAC protocols can lead to unnecessary retransmissions, idle listening, and slot wastage, thereby increasing power drain and reducing network lifetime^6^. Prior works have attempted to address these challenges through coordinated multi-channel schemes^7^, cooperative channel reservation^8^, and adaptive MAC mechanisms with cross-layer designs^9,10^. However, a persistent drawback in contention-free MAC models is the underutilization of allocated slots when vehicles generate smaller or less frequent data traffic^1^. This limitation becomes more critical in mixed traffic environments, where data transmission requirements vary significantly across vehicles due to differences in application priority, buffer occupancy, and energy states.

To overcome these inefficiencies, recent research has focused on adaptive and context-aware MAC protocols. For example, Abbas et al. proposed a priority-based MAC model for emergency communication in UAV-assisted VANETs^11^, Ahmad et al. introduced the HVMAC protocol with post-quantum authentication^12^, and Prathima et al. presented the IMAC-ELI framework combining adaptive contention management with stochastic routing^10^. These studies emphasize the importance of throughput, reliability, and security in VANETs, but efficient slot utilization under varying traffic loads remains an unresolved problem.

In earlier work, Tushar et al. proposed the Adaptive Collision-Free MAC Bit-Map Assisted (ACFM-BMA) protocol^1^, which integrated EV battery levels and buffer status into the scheduling process. By mapping vehicles’ real-time energy and buffer states, ACFM-BMA improved channel utilization, reduced idle listening, and achieved up to 45% energy savings under high traffic scenarios. However, a key limitation was identified: some devices underutilized their allocated slots when transmission demand was lower than the slot duration. This mismatch resulted in partial slot wastage, thereby reducing overall channel efficiency despite the improved prioritization.

To address this drawback, we now propose the Adaptive Collision-Free MAC Adaptive Bit-Map Assisted (ACFM-ABMA) protocol, which introduces slot fragmentation to further improve energy efficiency and channel utilization. In this approach, each allocated slot is divided into four subslots, with vehicles indicating their required fraction using two additional control bits. For example, a control field of “00” denotes the need for 25% of the slot, while “11” indicates full-slot utilization. This fine-grained allocation ensures that vehicles transmit data according to their actual buffer requirements, thereby minimizing channel wastage and reducing unnecessary energy expenditure. Moreover, the adaptive subslot mechanism enhances fairness by allowing more vehicles to access the channel simultaneously under high traffic conditions, improving scalability.

The novelty of the proposed ACFM-ABMA lies in its holistic integration of *battery status, buffer occupancy, and adaptive subslot allocation* within a contention-free MAC framework. Unlike existing slot-based or hybrid designs^7,13^, the proposed method dynamically adjusts resource allocation to match heterogeneous data traffic patterns, thereby balancing throughput, energy efficiency, and delay performance. Furthermore, the design is evaluated across low, moderate, and high traffic scenarios, considering parameters such as vehicle density, periodic monitoring traffic, and event-driven traffic probability. The results demonstrate that ACFM-ABMA achieves superior energy efficiency and throughput compared to both ACFM-BMA and conventional TDMA-based MAC models.

In summary, the contributions of this work are threefold:

1. Systematic identification and analytical characterization of slot underutilization is identified in existing bit-map assisted and TDMA-based MAC protocols for heterogeneous VANET traffic conditions.
2. A novel subslot fragmentation mechanism is introduced by adopting adaptive bit-mapping for efficient slot utilization.
3. Mathematical model is proposed and analytical parametric analysis is performed for the proposed ACFM-ABMA method.
4. Comprehensive performance analysis is performed across varying traffic densities, validating the protocol’s effectiveness in energy savings, channel utilization, and scalability.

The remainder of the paper is organized as follows. Section “Related works” reviews the related work on VANET MAC protocols, with emphasis on energy-efficient and adaptive slot allocation techniques. Section "System model and proposed methodology" presents the methodology of the proposed ACFM-ABMA protocol, detailing its frame structure and energy consumption model. Section "Result analysis and discussion" provides simulation-based performance analysis across diverse traffic scenarios, and Section “Conclusion” concludes the work with key findings and future directions.

## Related works

The design of efficient and adaptive Medium Access Control (MAC) protocols for Vehicular Ad Hoc Networks (VANETs) has attracted considerable attention over the past decade. Traditional contention-based protocols such as IEEE 802.11p and CSMA/CA offer simplicity, but they experience high collision probability, unpredictable delay, and low scalability under dense vehicular traffic conditions. These drawbacks motivated the development of hybrid and contention-free MAC schemes that incorporate scheduling, priority handling, and energy-efficient mechanisms to improve performance.

Although significant advancements are reported in priority-aware, energy-efficient, and multi-channel MAC designs, most existing works address these aspects independently. Very limited attention has been given to jointly optimizing slot utilization efficiency, buffer-aware prioritization, and energy states within a centralized TDMA-based VANET framework. In particular, fine-grained slot fragmentation for heterogeneous traffic demands remains underexplored. This gap motivates the proposed ACFM-ABMA protocol, which integrates adaptive sub-slot reservation with battery and buffer-aware scheduling.

Several studies have addressed priority-based communication in VANETs. Abbas et al.^11^ proposed ECPM-UAV, a UAV-assisted VANET model with priority-based MAC for emergency communication. Similarly, Zang et al.^2^ presented a cross-layer priority-based design using full-duplex technology for real-time emergency message dissemination. While both schemes improve delay and reliability, they do not explicitly consider slot utilization inefficiency caused by heterogeneous traffic demands.

Energy-efficient designs have also been investigated extensively. Shi et al.^5^ developed PAODV, a power-controlled routing mechanism at the MAC layer to reduce interference and energy consumption. Bhosale and Raisinghani^6^ modeled throughput-energy tradeoffs in IEEE 802.11-based networks, while Sharma et al.^14^ optimized AODV routing for IEEE 802.11g VANETs. These works confirm the importance of energy conservation but focus more on routing and link-level optimizations rather than slot-level allocation in MAC protocols.

**Table 1:** Comparison of related MAC protocols in VANETs.

Work	Approach	Key Strengths	Limitations
Abbas et al. (2025)^11^	Priority-based MAC with UAV support	Enhances emergency communication; improves data success rate and energy efficiency	Focused only on emergency traffic; no adaptive slot utilization
Zang et al. (2020)^2^	Full-duplex cross-layer priority design	Reduces collision probability; guarantees emergency message delivery	Requires FD hardware; no energy-awareness
Shi et al. (2010)^5^	Power-aware routing (PAODV)	Reduces interference and energy usage	Routing-focused; no MAC slot optimization
Bhosale and Raisinghani (2014)^6^	802.11 throughput-energy modeling	Provides empirical throughput-energy tradeoffs	Static analysis; lacks adaptive scheduling
Sharma et al. (2013)^14^	Optimized AODV with IEEE 802.11g	Improves energy efficiency and QoS	Routing-based; not applicable to MAC slot allocation
Kim et al. (2016)^4^	RSU-coordinated multi-channel MAC	Reduces broadcast collisions; increases throughput	No slot fragmentation or energy-aware scheduling
Li et al. (2015)^7^	RSU synchronous multi-channel reservation	Higher throughput and reduced congestion	Requires continuous RSU coordination
Yuan et al. (2012)^8^	Cooperative channel reservation (A-CCRM)	Adaptive power control and carrier sensing	Complex reservation overhead; no buffer/battery awareness
Compta et al. (2014)^13^	Network coding-based MAC (NCMOB-MAC)	Improves mobility support and throughput	Complexity; lacks energy and buffer considerations
Prathima et al. (2025)^10^	Adaptive MAC with stochastic routing	Increases packet delivery and energy efficiency	Focused on routing overhead; not fine-grained slot allocation
Anitha et al. (2025)^9^	Congestion-aware multihop routing with CSO	Reduces congestion and delay	Optimization-heavy; no direct MAC slot strategy
Ahmad et al. (2025)^12^	Hybrid MAC with PQWAA authentication	High throughput, delay reduction, and security	Security focus; no adaptive slot fragmentation
Chang et al. (2011)^15^	DORA fragmentation with rate adaptation	Improves goodput in noisy channels	Works for CSMA/CA only; not TDMA slot allocation
Khabazian et al. (2013)^16^	Analytical model for safety message broadcast	Reduces high-priority message delay	Limited to broadcast scenarios
Martín-Vega et al. (2018)^17^	Geolocation-based channel access (GLOC)	Optimizes reliability and capture probability	High computational cost; no buffer/energy integration
Our Work (ACFM-ABMA)	Adaptive bit-map assisted MAC with subslot fragmentation	Integrates battery, buffer, and slot occupancy with adaptive scheduling; reduces wastage and improves energy efficiency	Added complexity in scheduling; requires extra control bits

Multi-channel MAC strategies have been widely studied for efficient utilization of control and service channels. Kim et al.^4^ proposed C-MAC, an RSU-coordinated contention-free broadcasting method, while Li et al.^7^ introduced a synchronous multi-channel MAC scheme with rendezvous management. Yuan et al.^8^ presented A-CCRM with adaptive channel reservation, whereas Compta et al.^13^ employed network coding to enhance throughput and mobility support. These protocols improve throughput and delay but lack explicit integration of energy states and buffer information.

Recent adaptive and intelligent schemes further enhance VANET performance. Prathima et al.^10^ proposed IMAC-ELI, an adaptive MAC protocol integrated with stochastic route optimization, while Anitha et al.^9^ introduced a congestion-aware optimization using Chicken Swarm Optimization for multihop VANETs. Ahmad et al.^12^ developed HVMAC with post-quantum authentication, offering both reliability and security. Chang et al.^15^ proposed DORA, a fragmentation and rate adaptation method for noisy CSMA/CA channels, and Khabazian et al.^16^ analyzed safety message broadcasting delays. Martín-Vega et al.^17^ extended geolocation-based access using stochastic geometry to optimize capture probability and reliability. Recent studies have explored adaptive and energy-efficient MAC protocols across different wireless domains. Din *et al.*^18^ employed Q-learning to improve MAC convergence and energy efficiency, while Gómez^19^ proposed a mobility-adaptive slotted ALOHA scheme for vehicular networks. Dutta *et al.*^20^ addressed decentralized TDMA scheduling using multi-agent bandit learning, and Jayaweera *et al.*^21^ demonstrated time-slotted cooperative awareness in UAV networks. However, these works do not address fine-grained slot fragmentation and energy-aware reservation in centralized TDMA-based VANET MAC protocols, which is the focus of this study. To systematically position the proposed contribution, Table 1 compares representative MAC schemes in terms of slot utilization strategy, energy-awareness, and scheduling granularity. Table 1 also summarizes the major contributions of these works, highlighting their focus areas and limitations.

From the comparison, it is evident that while prior works have explored emergency prioritization, multi-channel coordination, energy-efficient routing, and congestion control, none explicitly integrate fine-grained slot fragmentation with battery-aware and buffer-aware reservation within a centralized TDMA scheduling framework. The proposed ACFM-ABMA protocol fills this gap by introducing a fine-grained fragmentation mechanism, supported by battery and buffer information, to maximize channel utilization and energy conservation across varying traffic conditions.

## System model and proposed methodology

**Fig. 1:**
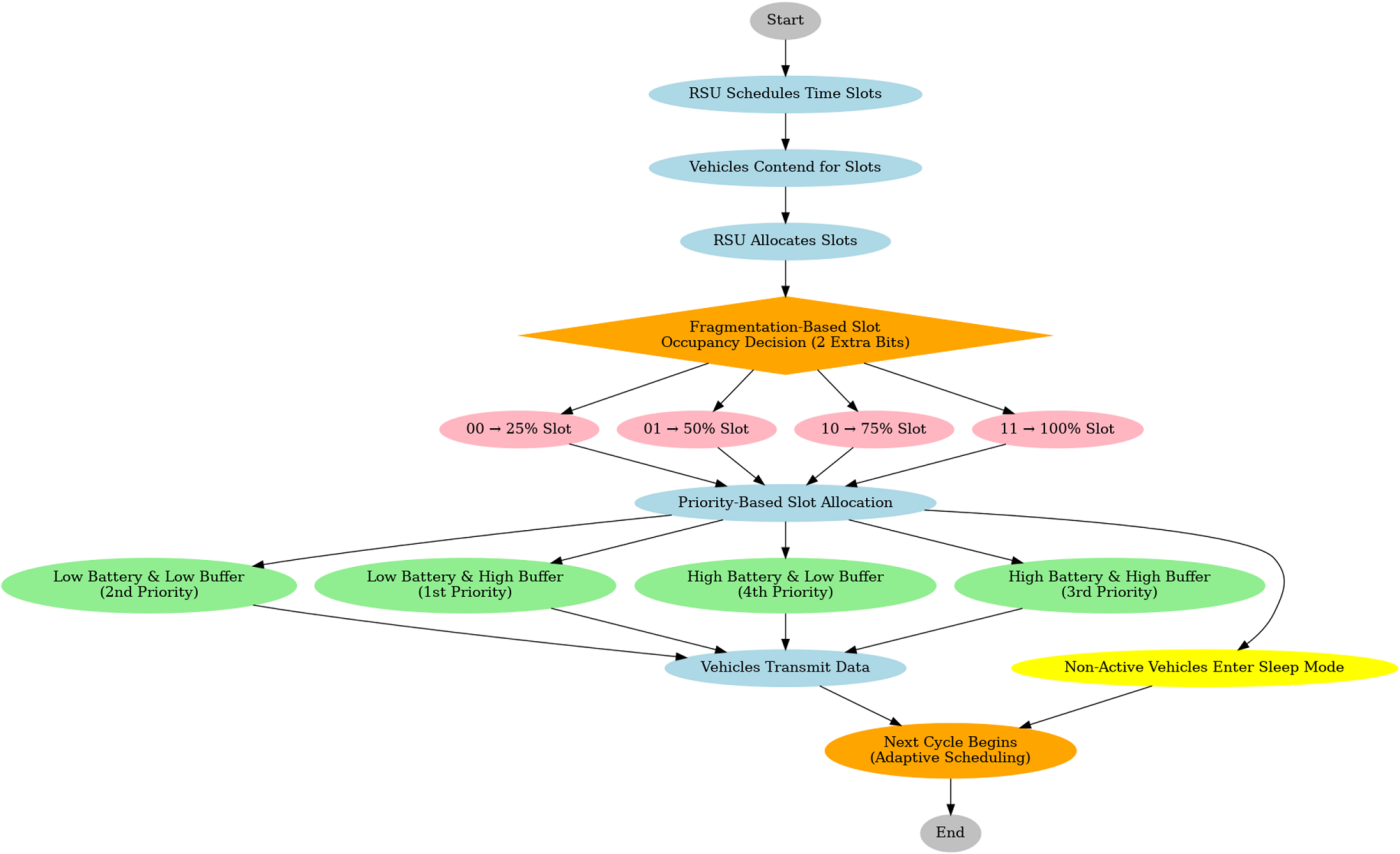
Flowchart of proposed ACFM-ABMA MAC protocol.

The system model of the proposed ACFM-ABMA protocol is illustrated in Fig. 1. The process begins with the RSU scheduling and allocating time slots, followed by a contention phase where vehicles express their transmission requests. To overcome slot underutilization, a fragmentation-based slot occupancy mechanism is introduced using two additional control bits, allowing each vehicle to request only 25%, 50%, 75%, or 100% of a slot based on its data requirements. Once the slot size is determined, a priority-based allocation is performed according to the vehicle’s battery level and buffer status, ensuring that nodes with critical energy or high buffer loads are served first. Active vehicles then transmit data in their allocated slots, while non-active vehicles conserve energy by entering sleep mode. The RSU collects feedback and initiates the next adaptive scheduling cycle, thereby achieving efficient channel utilization, reduced idle listening, and improved energy conservation across varying traffic conditions.

The proposed ACFM-ABMA protocol is readily implementable in both DSRC and 5G-V2X environments, as it operates purely at the MAC layer without requiring physical layer modifications. In DSRC, RSU beaconing enables centralized TDMA scheduling, while in 5G-V2X Mode-3, the bitmap-assisted reservation and sub-slot allocation can be mapped to gNB-assisted sidelink resource pools. This ensures backward compatibility and practical feasibility in existing vehicular communication infrastructures^22^.

In the present work the Medium Access Control (MAC) protocol is proposed for the Vehicular Ad-hoc Networks (VANETs), where time is discretized into units termed ’frames’. Each frame is an aggregate of a predetermined number of ’slots’, enabling structured temporal allocation for data transmission. The protocol operates on a cycle schedule maintained by Road Side Units (RSUs), with each cycle encompassing up to five frames ($F_{num} \le 5$), aligning with the beaconing interval standard of VANET applications, typically 100 milliseconds (ms).

Each frame is composed of one RSU slot ($T_{R}=T_{c}+G_s$), lasting 2 ms, followed by 36 data slots ($T_{ds}$), each with a duration of 0.5 ms. The cumulative cycle duration does not exceed 100 ms. Consequently, a full cycle can contain a maximum of five RSU slots and 180 data slots. In a dense traffic scenario, there will be 156 vehicles, which is lower than the available 180 data slots, ensuring that the system can allocate sufficient resources, similar to the ACFM-TDMA protocol in^23^.

The key distinction of ACFM-ABMA compared to ACFM-BMA lies in its adaptive slot fragmentation mechanism, which transforms fixed slot allocation into proportional resource assignment without altering frame duration or increasing control packet size. This enables fine-grained energy optimization while preserving MAC-layer compatibility.

### System assumptions

The following assumptions are considered for analytical tractability:

- All vehicles operate in a single-hop RSU coverage region.
- Perfect synchronization is assumed within each scheduling cycle.
- Packet size is bounded by 200 Bytes.
- Traffic consists of periodic ($m_v$) and event-driven ($n_v$) nodes.
- Channel errors and retransmissions are not considered.

### Data slot duration

**Fig. 2:**
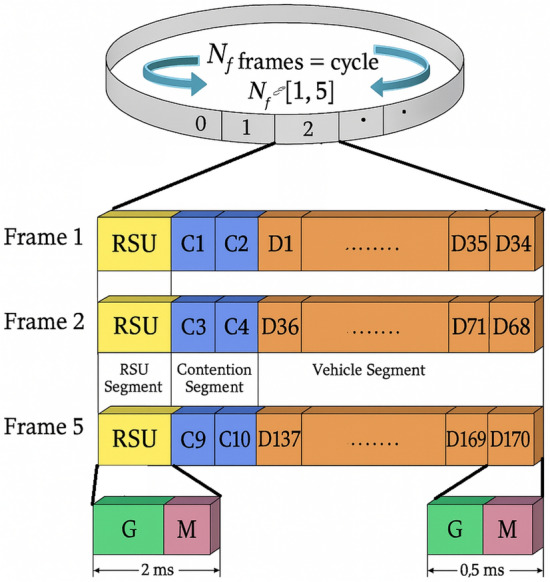
Frame structure of bit-mapping based MAC.

The data slot duration $T_{ds}$ must be configured to allow the transmission of a complete beacon packet. The frame structure of the MAC data transmission is shown in Fig. 2

1 $$\begin{aligned} T_{ds} = \frac{(P_{len} \times 8)}{R_d} + G_s \end{aligned}$$

Where $P_{len}$ is the beacon packet length ($\le 200$ Bytes), $R_d$ is the data rate (up to 4 Mbps), and $G_s$ is the guard interval (100 $\mu$s). Thus, $T_{ds}=0.5$ ms.

### RSU control packet size

The control packet is transmitted by the RSU unit. As per the standard, the maximum packet size of RSU control message can be derived as given in Eq 2:

2 $$\begin{aligned} P_{ctrl} = \frac{1}{8}(T_{R}-G_s)\times R_d \end{aligned}$$

With $T_{R}=2$ ms, $R_d=4$ Mbps, and $G_s=100 \mu$s, we get $P_{ctrl}\approx 950$ Bytes.

### Energy consumption analysis

The energy consumption analysis is explained for the proposed model and the existing model.

#### Energy model of ACFM-TDMA

As per the frame structure, the frame structure of the proposed TDMA MAC is divided into contention period and data transmission period. The energy consumed at the RSU for control packet transmission during contention period is derived as in Eq 3:

3 $$\begin{aligned} E^{RSU}_{TD-CP} = P_{tx}(T_{c}+G_s) \times N_v \end{aligned}$$

Eq 4 models the vehicle-side energy consumption during control packet reception in ACFM-TDMA, accounting for radios turning on across successive frames as slots are allocated. It expresses the cumulative reception cost in terms of frame count, number of vehicles, and control slot duration^23^. The detailed explanation is discussed in^1^. The energy consumed at the vehicles for control packet reception is:

4 $$\begin{aligned} \begin{aligned} E^{V}_{TD-CP}&= P_{rx}(T_{c}+G_s)\Big (36\frac{F_{num}(F_{num}-1)}{2}+(N_v-36(F_{num}-1))F_{num}\Big ) \\&= P_{rx}(T_{c}+G_s)\Big (F_{num}(N_v-18(F_{num}-1))\Big ) \end{aligned} \end{aligned}$$

Eq 5 represents the total energy consumed by vehicles during the data slot phase in ACFM-TDMA. The first term captures the energy of active vehicles ($m_v+n_v$) that both transmit and keep idle radios listening, while the second term accounts for the idle listening cost of the remaining non-active vehicles across their slots^23^.The RSU energy consumption for data slot reception is:

5 $$\begin{aligned} \begin{aligned} E^{RSU}_{TD-DS}&= n_vP_{rx}(T_{ds}+G_s)+m_vP_{rx}(T_{ds}+G_s)+\\&(N_v-m_v-n_v-1)P_{idle}(T_{ds}+G_s) \end{aligned} \end{aligned}$$

Eq 6$E^{V}_{TD-DS}$ represents the total energy consumed by vehicles during the data slot phase in the ACFM-TDMA protocol. It is composed of two main parts. The first part corresponds to the energy expended by the active vehicles, denoted as $(m_v+n_v)$, which not only consume transmission energy while sending their packets but also incur idle energy consumption while waiting in other vehicles’ slots. The second part accounts for the idle listening cost of the remaining non-active vehicles, expressed as $(N_v-m_v-n_v-1)$, which keep their radios active in idle mode throughout the slots allocated to other vehicles. Together, this formulation highlights the dual contribution of both transmitting and non-transmitting nodes to the overall vehicular energy consumption, emphasizing the significant role of idle listening in the system’s energy inefficiency^23^. The vehicle-side energy consumption during data slots is:

6 $$\begin{aligned} E^{V}_{TD-DS} = (m_v+n_v)\Big (P_{tx}(T_{ds}+G_s)+(N_v-2)P_{idle}(T_{ds}+G_s)\Big ) + (N_v-m_v-n_v-1)(N_v-1)P_{idle}(T_{ds}+G_s) \end{aligned}$$

#### Energy model of ACFM-BMA

To improve the performance, tushar et al.^1^ proposed an energy-efficient bit-mapping method for the energy efficient data transmission. The author proposed that 2 bit status can be used for the battery and buffer status of the sensor node and slot allotment priority can be decided based on this bit-mapping. For ACFM-BMA, each frame reserves 2 contention slots, reducing the number of data slots to 34. The detailed explanation is discussed in^1^. The RSU control packet energy can be expressed as:

7 $$\begin{aligned} E^{RSU}_{BMA-CP} = P_{tx}(T_{c}+G_s)N_v \end{aligned}$$

Similar to the TDMA, the total energy consumption of ACFM-BMA for Vehicle-side control packet reception can be expressed as:

8 $$\begin{aligned} \begin{aligned} E^{V}_{BMA-CP}&= P_{rx}(T_{c}+G_s)\Big (34\frac{F_{num}(F_{num}-1)}{2}+(N_v-34(F_{num}-1))F_{num}\Big ) \\&= P_{rx}(T_{c}+G_s)\Big (F_{num}(N_v-17(F_{num}-1))\Big ) \end{aligned} \end{aligned}$$

The energy consumption in the proposed ACFM-BMA protocol can be divided into contention and data transmission phases. The RSU contention energy, given by $E^{RSU}_{BMA-CAP} = P_{rx}(2T_{ds}+G_s)F_{num}$, denotes the energy expended by the RSU for receiving contention packets during the contention access period across all frames.

9 $$\begin{aligned} E^{RSU}_{BMA-CAP} = P_{rx}(2T_{ds}+G_s)F_{num} \end{aligned}$$

Similarly, the vehicle contention packet energy, expressed as $E^{V}_{BMA-CAP} = N_vP_{tx}T_{co}$, captures the energy used by all $N_v$ vehicles while transmitting their two-bit bitmap control packets, where $T_{co}$ denotes the duration of this bitmap message.

10 $$\begin{aligned} E^{V}_{BMA-CAP} = N_vP_{tx}T_{co} \end{aligned}$$

where $T_{co}$ is the time for bit-map control packet.

For the data slot reception, the RSU energy is represented by, $E^{RSU}_{BMA-DS} = n_vP_{rx}(T_{ds}+G_s)+m_vP_{rx}(T_{ds}+G_s)+(N_v-m_v-n_v-1)P_{slp}(T_{ds}+G_s)$, which accounts for the RSU receiving energy from event-driven nodes ($n_v$) and continuous traffic nodes ($m_v$), while the remaining vehicles consume sleep-mode energy.

11 $$\begin{aligned} E^{RSU}_{BMA-DS} = n_vP_{rx}(T_{ds}+G_s)+m_vP_{rx}(T_{ds}+G_s) + (N_v-m_v-n_v-1)P_{slp}(T_{ds}+G_s) \end{aligned}$$

On the other hand, the vehicle data slot transmission energy is given by $E^{V}_{BMA-DS} = (m_v+n_v)\Big (P_{tx}(T_{ds}+G_s)+(N_v-2)P_{idle}(T_{ds}+G_s)\Big )$, representing the energy consumed by active vehicles during transmission, along with the idle listening power when not transmitting^23^.

12 $$\begin{aligned} E^{V}_{BMA-DS} = (m_v+n_v)\Big (P_{tx}(T_{ds}+G_s)+(N_v-2)P_{idle}(T_{ds}+G_s)\Big ) \end{aligned}$$

#### Energy model of ACFM-ABMA (proposed)

As discussed, in the existing methods, many of the times devices not utilizes complete slot due to non-uniformity and abruptness of data generation. Therefore, in the proposed ACFM-ABMA protocol, each data slot $T_{ds}$ is fragmented into four subslots, and vehicles indicate their slot requirement using 2 extra bits during contention period slot reservation:

- 00: 25% of slot ($$0.25T_{ds}$$)
- 01: 50% of slot ($$0.5T_{ds}$$)
- 10: 75% of slot ($$0.75T_{ds}$$)
- 11: 100% of slot ($$T_{ds}$$)

Vehicle reserves a slot based on the requirement during contention access period. Both vehicle and RSU turns on Radio only for the reserved subslot duration. Thus, reduces the energy consumption for both the vehicle and RSU.

The contention slot duration remain unchanged as used in BMA. RSU control packet energy remains the same as BMA:

13 $$\begin{aligned} E^{RSU}_{ABMA-CP} = P_{tx}(T_{c}+G_s)N_v \end{aligned}$$

Similarly, the Vehicle reception energy for the contention period is also remain unchanged as derived for BMA. The vehicles energy consumption for the contention period duration is given by:

14 $$\begin{aligned} E^{V}_{ABMA-CP} = P_{rx}(T_{c}+G_s)\Big (F_{num}(N_v-\alpha (F_{num}-1))\Big ) \end{aligned}$$

where $\alpha =17$ (for 34 data slots/frame).

As discussed during CAP, in the proposed method, each device transmit 2 bits extra for the subslot requirement. However, overall size of control packet remain unchanged. Therefore, the total energy consumption during RSU contention accesss period is given by:

15 $$\begin{aligned} E^{RSU}_{ABMA-CAP} = P_{rx}(2T_{ds}+G_s)F_{num} \end{aligned}$$

The total energy consumption for the vehicle during contention access period is given by:

16 $$\begin{aligned} E^{V}_{ABMA-CAP} = N_vP_{tx}T_{c} \end{aligned}$$

In the proposed ACFM-ABMA protocol, slot fragmentation is introduced to optimize channel utilization and reduce idle energy consumption. The RSU reception energy is expressed as:

17 $$\begin{aligned} E^{RSU}_{ABMA-DS} = \sum _{i=1}^{N_v} P_{rx}\big (\beta _iT_{ds}+G_s\big ) + (N_v-N_{active})P_{slp}(T_{ds}+G_s) \end{aligned}$$

where $\beta _i \in \{0.25,0.5,0.75,1\}$ indicates the fraction of the slot actually used by the *i*-th vehicle, and $N_{active}$ represents the number of vehicles with data to transmit. This formulation shows that the RSU only spends reception energy proportional to the fraction of the slot occupied, while the non-active vehicles consume minimal energy in sleep mode. The proposed slot fragmentation mechanism introduces a fixed overhead of only two additional control bits per vehicle to indicate sub-slot requirements. This overhead is embedded within the existing bitmap control message, leaving the control packet size, contention duration, and frame structure unchanged.

The vehicle-side transmission energy is given by:

18 $$\begin{aligned} E^{V}_{ABMA-DS} = \sum _{i=1}^{N_{active}} \Big (P_{tx}(\beta _iT_{ds}+G_s) + (N_v-2)P_{idle}(T_{ds}+G_s)\Big ) \end{aligned}$$

which represents the energy spent by active vehicles when transmitting data during their allocated subslots, in addition to the idle listening cost during other vehicles’ transmissions. By scaling slot usage through the factor $\beta _i$, ACFM-ABMA minimizes underutilized time and reduces unnecessary energy consumption, offering finer-grained efficiency compared to conventional BMA schemes^23^. Thus, ACFM-ABMA optimizes energy usage by reducing idle listening and ensuring vehicles only activate radios for the fraction of the slot they require, leading to finer granularity in CFP energy conservation compared to ACFM-BMA.

### Computational complexity analysis

The scheduling complexity of ACFM-ABMA at the RSU is O($N_v$), since slot assignment requires a single-pass prioritization based on battery, buffer, and fragmentation status. The additional overhead introduced by sub-slot fragmentation is constant (2 bits per vehicle) and does not increase frame duration or contention length. Therefore, the proposed scheme preserves linear scalability with respect to network size.

### Average latency analysis

The average latency analysis is explained for the proposed model and the existing model. In order to analytically capture the delay characteristics of the considered MAC protocols, the average latency is defined as the expected waiting time experienced by a packet from its generation instant to the start of its allocated transmission opportunity in the subsequent scheduling cycle. Since all schemes operate under a centralized, contention-free TDMA framework coordinated by the RSU, latency is deterministic and bounded by the scheduling cycle duration.

#### Average latency of ACFM-TDMA

For the baseline ACFM-TDMA protocol, each vehicle is assigned a fixed data slot irrespective of its actual traffic demand. Hence, the average latency experienced by a packet in the arbitrary cycle can be expressed as

19 $$\begin{aligned} L_{\text {TDMA}} = \frac{T_c + N_v T_{ds}}{n_v + m_v}, \end{aligned}$$

where $T_c$ denotes the RSU control duration, $T_{ds}$ is the fixed data slot duration, $N_v$ is the total number of vehicles, and $(n_v+m_v)$ represents the number of active vehicles generating event-driven and periodic traffic, respectively. The expression highlights that latency grows linearly with network size due to rigid full-slot allocation and idle waiting.

#### Average latency of ACFM-BMA

In the ACFM-BMA protocol, additional contention and bitmap exchange overhead is introduced to improve energy efficiency, but full-slot allocation is still preserved. Consequently, the average latency becomes

20 $$\begin{aligned} L_{\text {BMA}} = \frac{T_c + N_v T_c + (m_v+n_v) T_{ds}}{n_v + m_v}, \end{aligned}$$

where the second term $N T_c$ accounts for bitmap-assisted contention overhead. Although ACFM-BMA reduces idle listening, the latency improvement remains limited due to the absence of fine-grained slot utilization.

#### Average latency of ACFM-ABMA

For the proposed ACFM-ABMA protocol, slot fragmentation enables vehicles to occupy only a fraction of a slot proportional to their buffer demand. Let $\beta$ denote the average slot utilization factor for moderate, worst-case, and best-case traffic distributions. The average latency in the arbitrary cycle is therefore given by

21 $$\begin{aligned} L_{\text {ABMA}_m} = \frac{T_c + N_v T_c + (m_v+n_v) \beta T_{ds}}{\beta (n_v+m_v)}, \end{aligned}$$

The above expressions clearly demonstrate that, unlike ACFM-TDMA and ACFM-BMA, the proposed ACFM-ABMA scales latency with the effective slot utilization factor.

**Table 2:** Analytical comparison of MAC protocols.

Metric	ACFM-TDMA	ACFM-BMA	ACFM-ABMA
Slot Utilization	Fixed (100%)	Fixed (100%)	Adaptive (25–100%)
Energy Saving	Low	Moderate	High
Idle Listening	High	Moderate	Minimal
Latency Scaling	O($N_v$)	O($N_v$)	O($\beta N_v$)
Control Overhead	None	Bitmap	Bitmap + 2 bits

Table 2 summarizes the analytical differences among TDMA, BMA, and the proposed ABMA protocol in terms of slot utilization, energy saving, latency scaling, and control overhead. It clearly highlights that ABMA achieves higher energy saving and improved scalability through adaptive slot fragmentation while maintaining minimal additional control overhead.

## Result analysis and discussion

The performance of the proposed ACFM-ABMA protocol is evaluated through MATLAB-based simulations and compared with the baseline ACFM-TDMA and ACFM-BMA protocols.The evaluation focuses on two primary performance metrics: (i) total energy consumption per cycle, including transmission, reception, idle, and sleep components, and (ii) average latency, defined as the expected waiting time from packet generation to scheduled transmission. These metrics allow direct comparison of slot utilization efficiency and scheduling effectiveness across TDMA, BMA, and ABMA. The simulation setup employs the parameters listed in Table 3, including power consumption values for transmission ($P_{tx}=0.891$ W), reception ($P_{rx}=0.0363$ W), idle mode ($P_{idle}=P_{rx}$), and sleep mode ($P_{slp}=0.01P_{rx}$). The time-related parameters are configured with an RSU segment duration of $T_c=1.9$ ms, data slot duration of $T_{ds}=0.4$ ms, and bitmap control duration of $T_{co}=20\,\mu$s, ensuring consistency with the IEEE 802.11p standard. The number of vehicles *N* is randomized between 10 and 150, while the number of frames is determined as $N_{Tc}=\lceil N/36 \rceil$ for ACFM-TDMA and $N_{BTc}=\lceil N/34 \rceil$ for ACFM-BMA/ABMA, reflecting the slot allocation structure. To represent diverse traffic scenarios, three probability distributions are considered: moderate load ($TF_m=0.4375$), biased load ($TF_b=0.325$), and worst-case load ($TF_w=0.925$). Each simulation is conducted for $l=100$ cycles to ensure statistical reliability. The detailed parameter table is given in Table 3.

**Table 3:** Simulation parameters for ACFM-ABMA evaluation.

Parameter	Symbol/Value	Description
Power Parameters		
Transmit Power	$P_{tx} = 3.3 \times 0.270 \,\text {W}$	Transmission power consumption
Receive Power	$P_{rx} = 3.3 \times 0.011 \,\text {W}$	Reception power consumption
Idle Power	$P_{idle} = P_{rx}$	Power demand in idle stage
Sleep Power	$P_{slp} = 0.01P_{rx}$	Power demand in sleep stage
Time Parameters		
RSU Segment Duration	$T_c = 1.9\,\text {ms}$	RSU control slot time (4 Mbps)
Bit-map Control Duration	$T_{co} = 20\,\mu \text {s}$	Bitmap transmission time
Data Slot Duration	$T_{ds} = 0.4\,\text {ms}$	Data packet transmission time
Traffic Fractions		
Moderate Load	$p_{01}=p_{02}=p_{03}=0.25,~p_{04}=0.25$	Equal probability of slot use
	$TF_m = 0.625$	Average slot utilization fraction
Biased Load	$p_{01}=0.7,~p_{02}=p_{03}=p_{04}=0.1$	Light slot use dominates
	$TF_b = 0.4$	Average slot utilization fraction
Worst Case	$p_{01}=p_{02}=p_{03}=0.1,~p_{04}=0.7$	Full slot use dominates
	$TF_w = 0.85$	Average slot utilization fraction
Simulation Setup		
Number of Vehicles	$N_v$	Randomized between different range
Frames (TDMA)	$N_{Tc} = \lceil N/36 \rceil$	Frames required in TDMA
Frames (BMA)	$N_{BTc} = \lceil N/34 \rceil$	Frames required in BMA/ABMA
Periodic Nodes	$m_v = 0.2N_v$	Nodes generating periodic traffic
Cycles	$l = 100$	Total simulation cycles

**Fig. 3:**
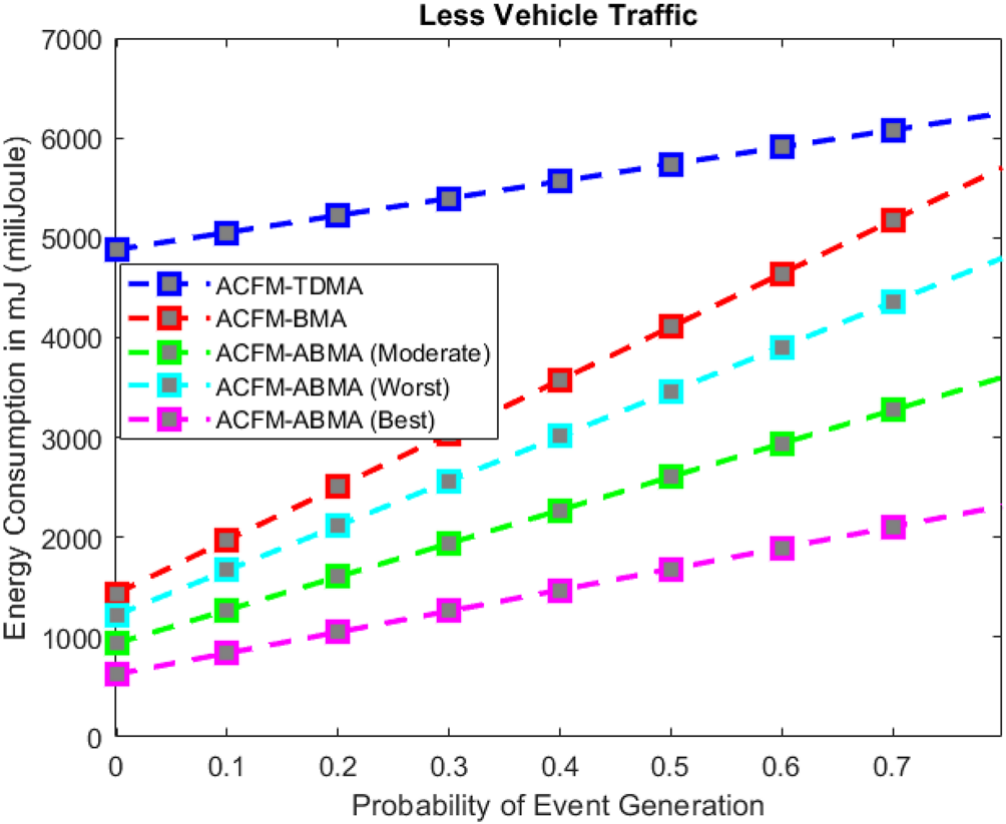
Energy consumption for varying event probability (low density).

**Fig. 4:**
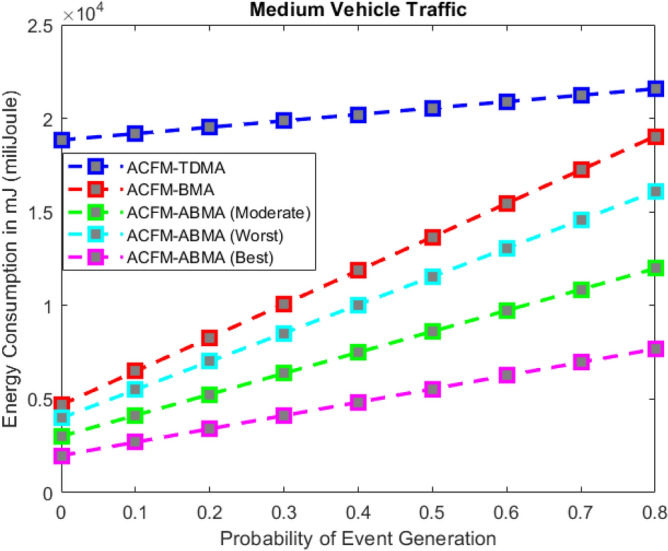
Energy consumption for varying event probability (medium density).

**Fig. 5:**
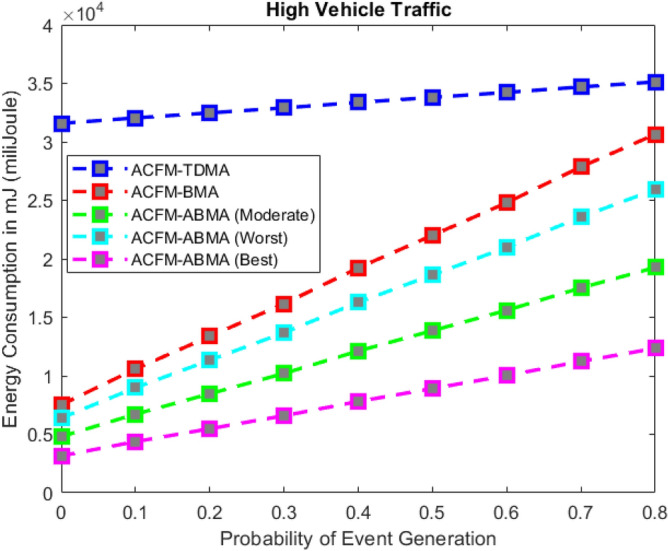
Energy consumption for varying event probability (high density).

### Scenario 1 – energy consumption for varying event probability

In Scenario 1, the impact of varying event generation probability on energy consumption was analyzed under low, medium, and high vehicular traffic densities as shown in Figs. 3, 4, and 5. The results show that in all cases, ACFM-TDMA incurs the maximum energy consumption due to fixed slot allocation, which forces most vehicles to remain in idle listening mode, even when they have no data to transmit. ACFM-BMA, by introducing contention-based bit-mapping, reduces idle listening cost, yet it suffers from slot underutilization when traffic load is light or uneven. By contrast, the proposed ACFM-ABMA consistently achieves lower energy consumption across all traffic densities, benefiting from slot fragmentation that allows vehicles to occupy only the fraction of a slot proportional to their transmission needs.

Specifically, under high vehicle traffic conditions, ABMA shows the steepest reduction in energy consumption compared to TDMA and BMA, with its best-case slot allocation nearly halving the energy demand at higher event probabilities. Even in the worst-case setting, where fragmentation is less favorable, ABMA still outperforms BMA. For low and medium traffic, the advantage of ABMA is most evident at higher event probabilities, where idle listening in TDMA and BMA rises significantly. These findings confirm that ACFM-ABMA is highly adaptive and energy-efficient under probabilistic event traffic, outperforming existing MAC variants across all load conditions.

### Scenario 2 – energy consumption for varying number of nodes

**Fig. 6:**
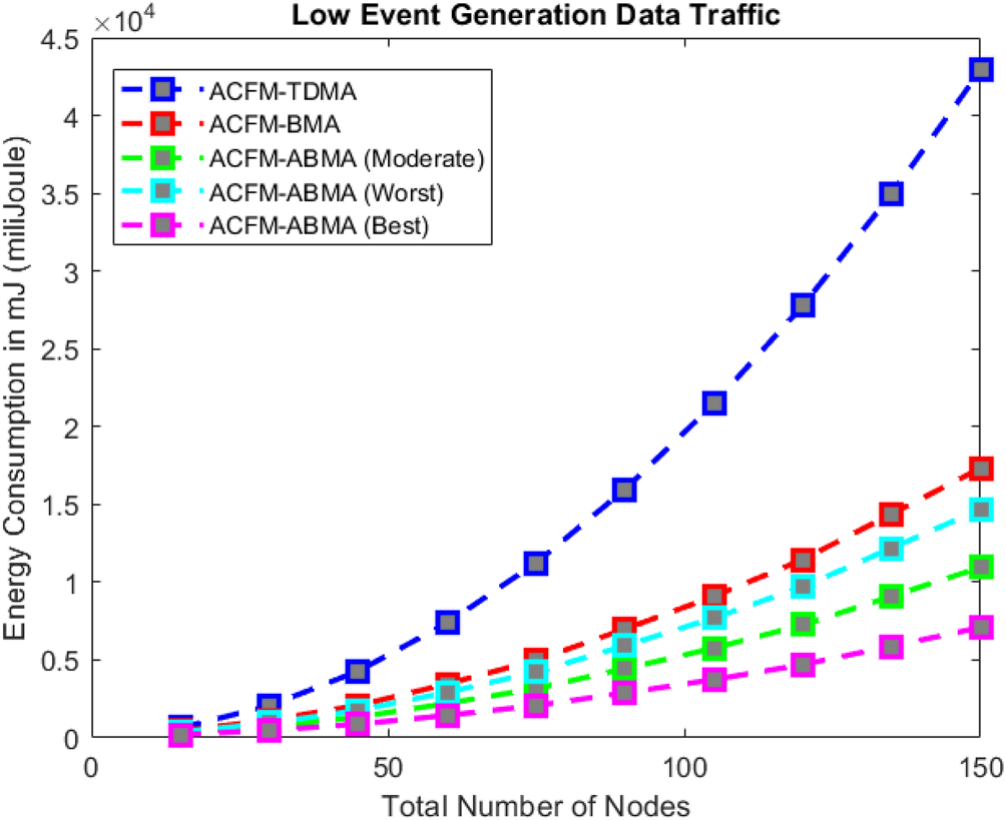
Energy consumption for varying vehicle density (low event probability).

**Fig. 7:**
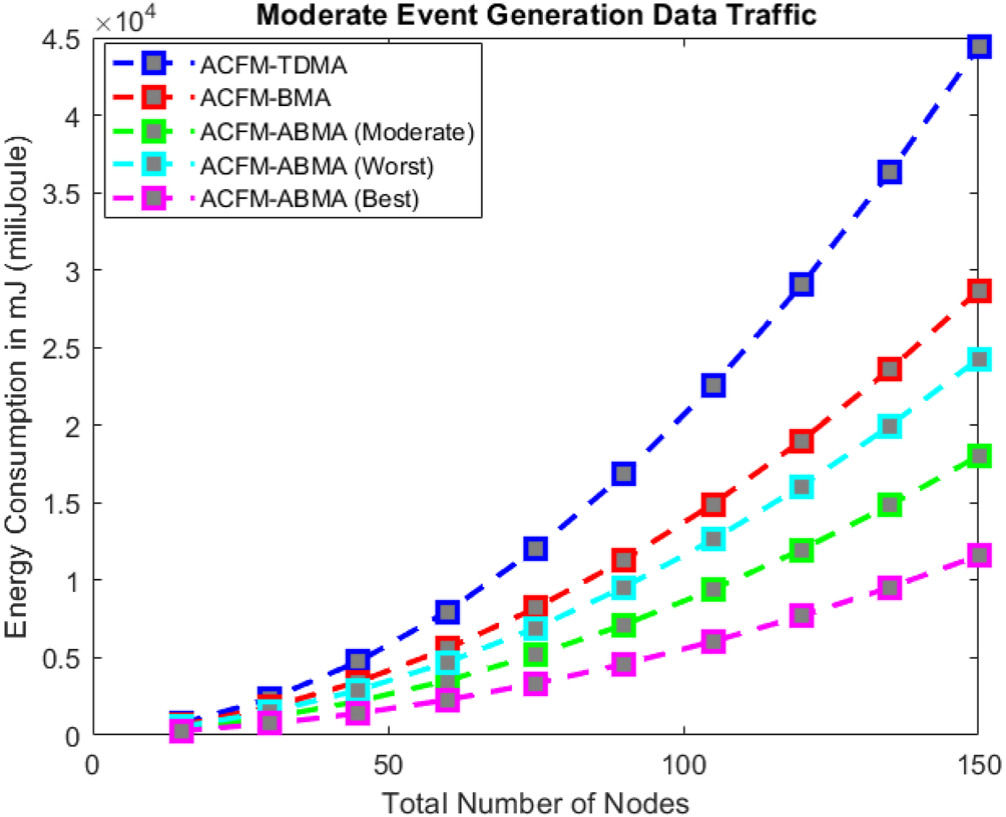
Energy consumption for varying vehicle density (medium event probability).

**Fig. 8:**
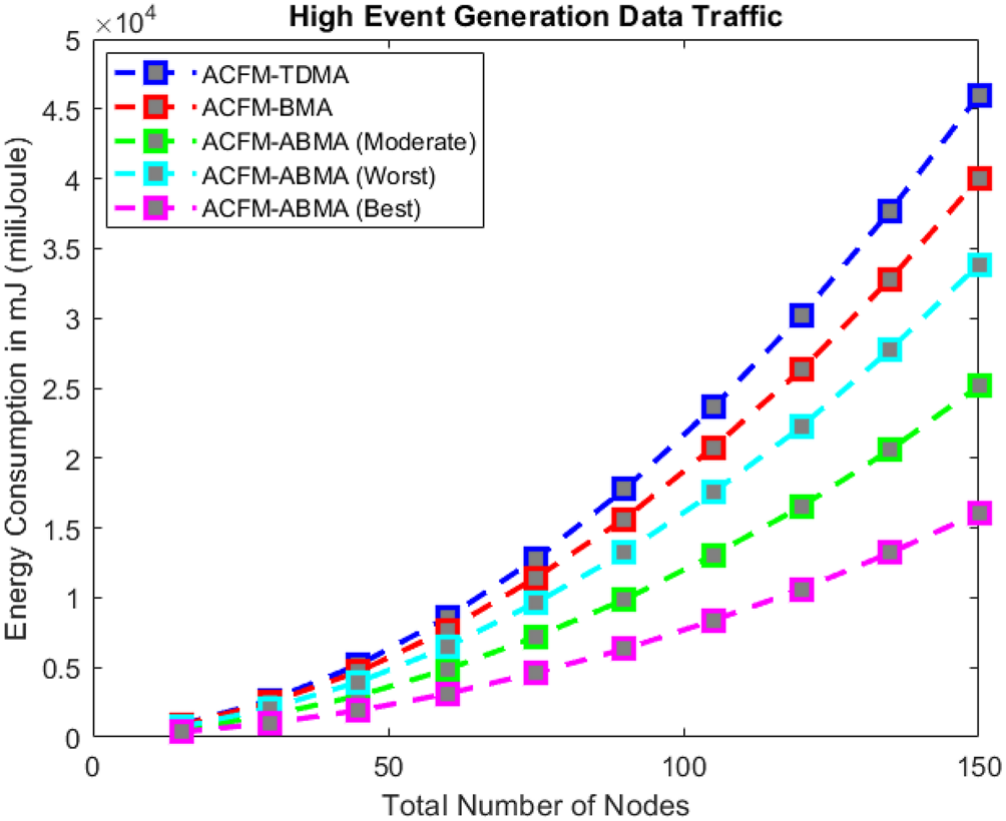
Energy consumption for varying vehicle density (high event probability).

Scenario 2 examines the effect of increasing the number of nodes on energy consumption under low, medium, and high event generation conditions as shown in Figs. 6, 7, and 8 respectively. With growing node density, ACFM-TDMA shows a sharp increase in energy usage due to the large number of vehicles forced to remain active during control and data phases. ACFM-BMA performs better by distributing slots through contention but becomes inefficient as contention overhead grows with network size. The proposed ACFM-ABMA demonstrates superior scalability, as slot fragmentation ensures that channel resources are allocated in finer granularity, preventing energy wastage and supporting larger vehicular densities efficiently. In addition, synchronization in ACFM-ABMA is maintained through RSU-coordinated beaconing and guard intervals, ensuring robustness against clock drift in dense networks. The bitmap-based deterministic scheduling scales linearly with node density, while slot fragmentation enhances scalability by accommodating heterogeneous traffic without increasing frame duration.

The results reveal that under high event generation traffic, ABMA achieves substantial energy savings compared to both BMA and TDMA, with its best-case performance yielding up to 40–50% lower energy consumption when the network reaches its maximum node count. For moderate and low event traffic, ABMA maintains steady energy efficiency, where even the worst-case fragmentation performs comparably to or better than BMA. This indicates that ABMA not only adapts to dynamic event probabilities but also scales effectively with network growth, maintaining predictable energy efficiency gains as vehicular density increases.

### Scenario 3 – energy consumption for varying periodic monitoring nodes

**Fig. 9:**
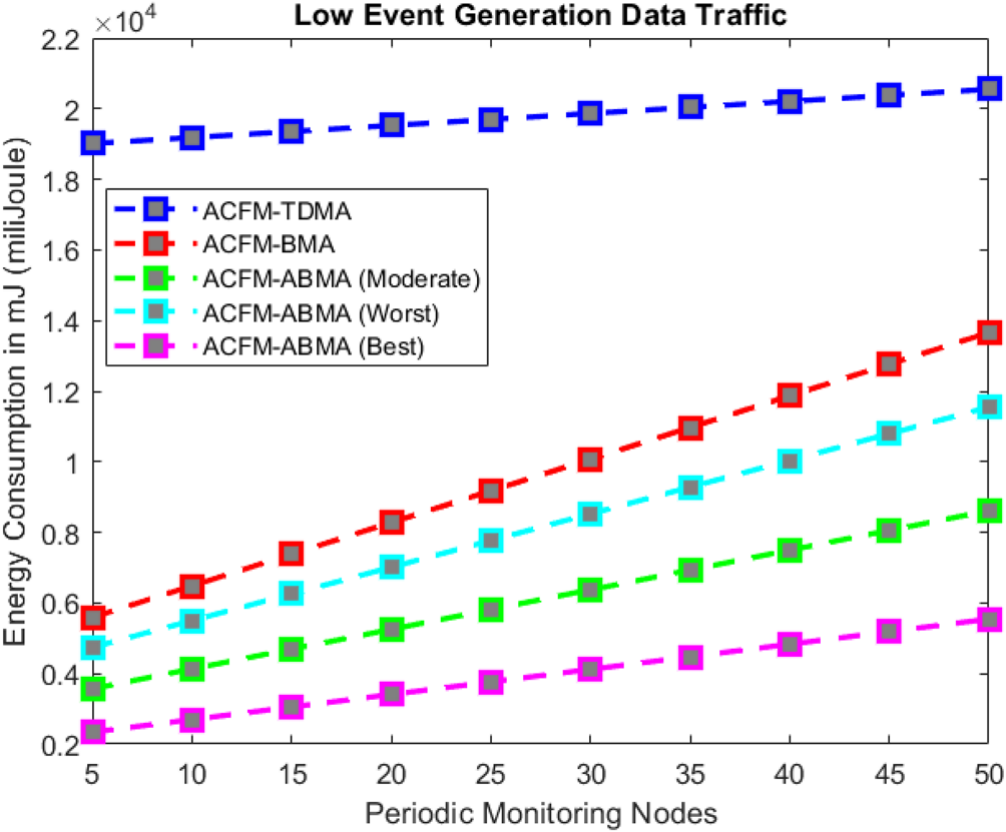
Energy consumption for varying periodic monitoring nodes (low event probability).

**Fig. 10:**
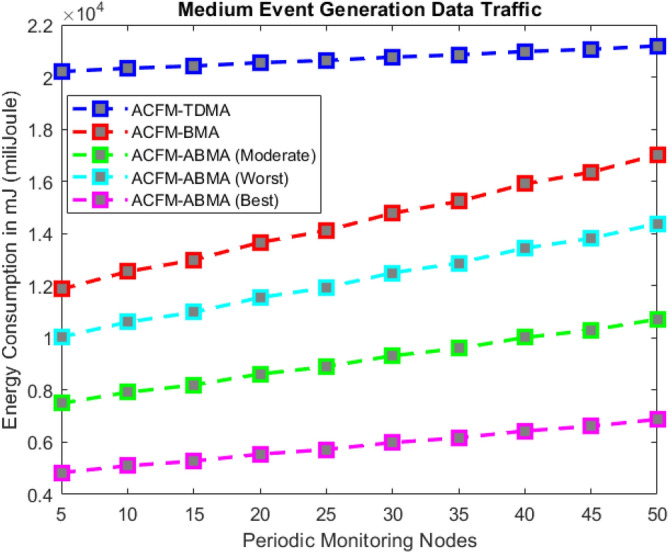
Energy consumption for periodic monitoring nodes (medium event probability).

**Fig. 11:**
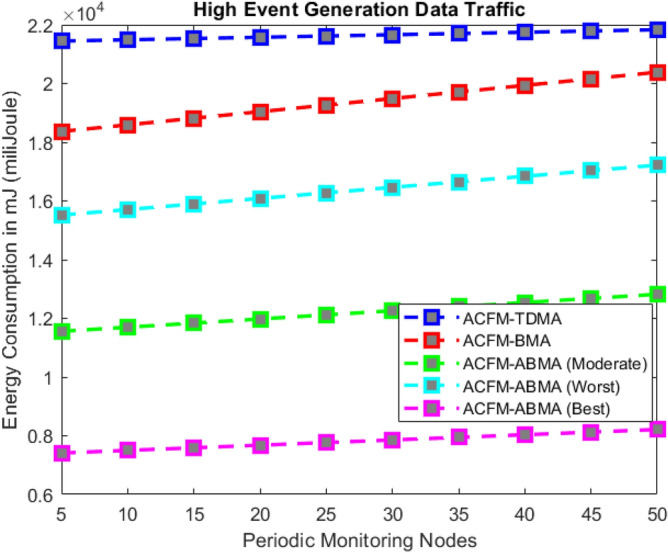
Energy consumption for varying periodic monitoring nodes (high event probability).

In Scenario 3, the focus is on the number of periodic monitoring nodes generating regular traffic under low, medium, and high event generation conditions as shown in Figs. 9, 10, and 11 respectively. For ACFM-TDMA, energy consumption remains consistently high since all vehicles remain active regardless of traffic type, amplifying idle listening costs. ACFM-BMA lowers energy consumption by bit-mapping, but the rigid full-slot allocation still leads to inefficiency when monitoring nodes generate small packets. The proposed ACFM-ABMA overcomes this drawback by allowing partial slot usage through fragmentation, ensuring that monitoring nodes transmitting low volumes of data consume only proportional energy.

The results confirm that under high event traffic, ABMA achieves the most notable energy efficiency, with its best-case operation consuming nearly one-third less energy than BMA and more than half less than TDMA. In low and medium event traffic, the performance advantage is even more pronounced, as ABMA significantly reduces unnecessary idle listening and slot underutilization. Across all cases, ABMA’s worst-case performance still outperforms BMA, demonstrating robustness and adaptability. These findings highlight that ABMA is highly effective in heterogeneous traffic environments, where a mix of periodic and event-driven data requires fine-grained scheduling to balance throughput and energy efficiency.

### Scenario 4 – latency analysis of varying number of nodes

In low event generation conditions, latency differences become more pronounced due to sparse traffic and smaller packet sizes. ACFM-TDMA continues to offer minimal latency because packets are transmitted in preassigned slots irrespective of demand; however, this advantage is misleading as most of the slot duration remains unused. ACFM-BMA experiences higher latency owing to contention and full-slot reservation even for small payloads. The proposed ACFM-ABMA outperforms BMA in both moderate and worst cases by significantly reducing waiting time through partial slot usage, which aligns with the analytical latency expressions incorporating the slot utilization factor. The best-case ABMA scenario shows higher latency due to a higher average utilization factor, but still remains within deterministic bounds. These results demonstrate that under low traffic loads, ACFM-ABMA efficiently balances latency and energy by eliminating unnecessary slot wastage while maintaining contention-free access.

**Fig. 12:**
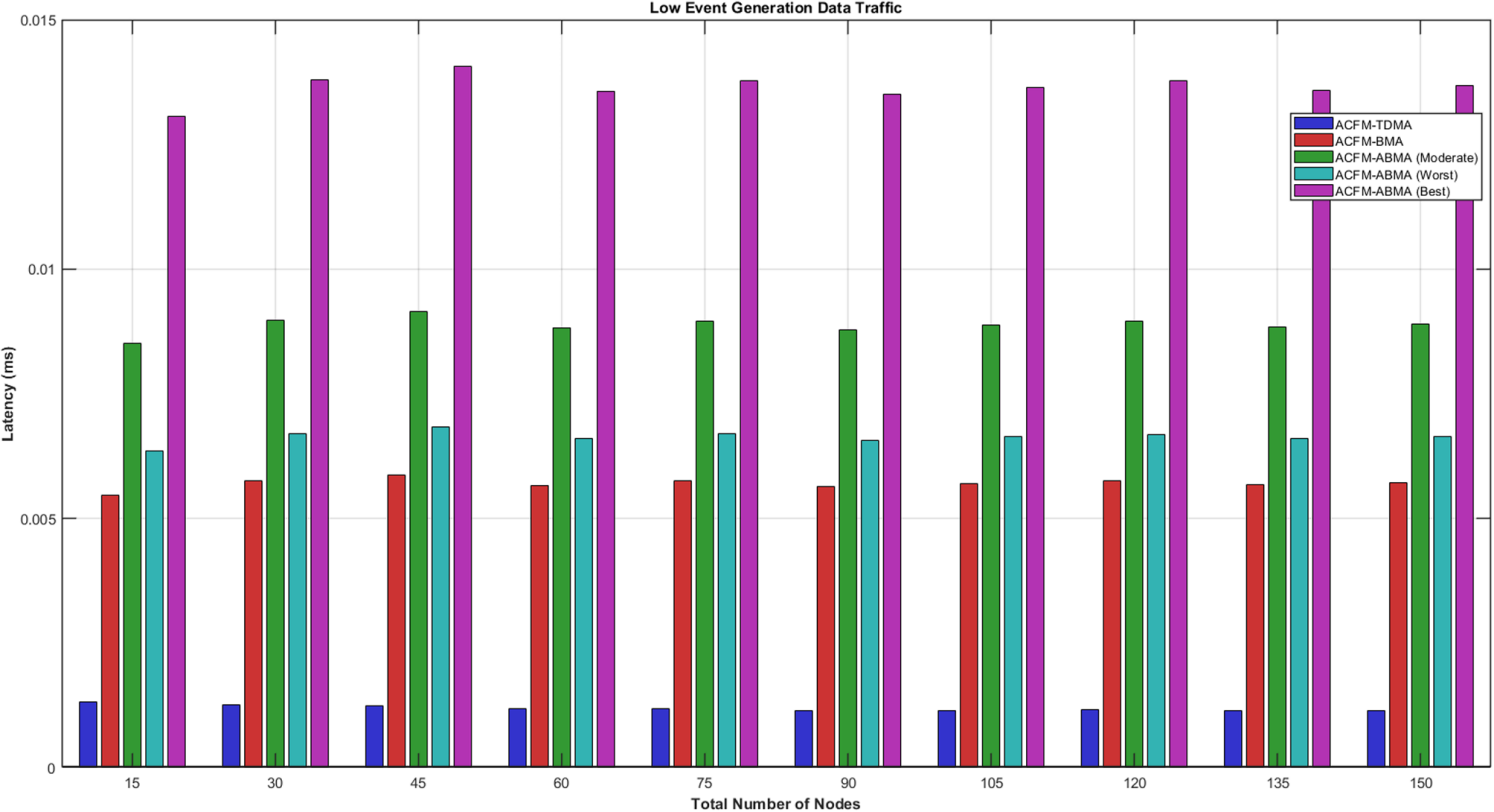
Energy consumption for varying vehicle density (low event probability).

Under moderate event generation traffic, the latency behavior clearly reflects the impact of reservation rigidity and slot utilization efficiency. ACFM-TDMA exhibits the lowest absolute latency because it allocates fixed slots deterministically without contention overhead; however, this comes at the cost of severe slot underutilization and high energy wastage, as discussed earlier. ACFM-BMA shows a noticeable increase in latency due to the additional bitmap-based contention overhead introduced during scheduling, although it improves energy efficiency compared to TDMA. The proposed ACFM-ABMA demonstrates differentiated latency behavior across best, worst, and moderate cases. In the moderate case, ABMA achieves lower latency than BMA due to reduced effective transmission time enabled by sub-slot fragmentation. The best-case ABMA scenario shows the highest latency values, which is expected since more nodes utilize larger slot fractions, increasing effective cycle duration. Overall, the results confirm that ACFM-ABMA maintains bounded and predictable latency while achieving superior utilization efficiency compared to existing schemes.

**Fig. 13:**
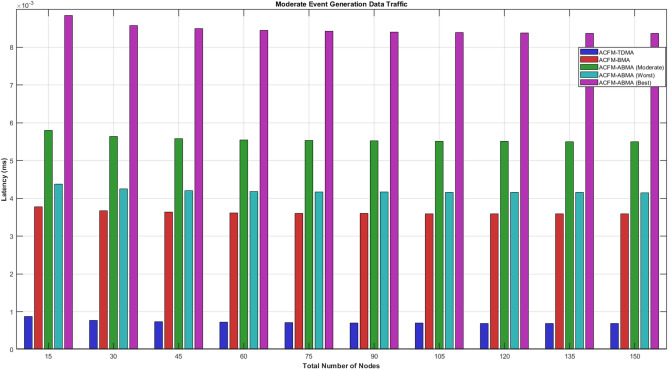
Energy consumption for varying vehicle density (medium event probability).

For high event generation traffic, all protocols experience increased latency due to heavier network load and a larger number of active nodes per cycle. ACFM-TDMA shows relatively stable latency growth because of its fixed scheduling, but this again leads to excessive idle listening and poor scalability. ACFM-BMA incurs additional latency due to bitmap contention overhead, which becomes more significant as traffic intensity increases. In contrast, the proposed ACFM-ABMA exhibits superior adaptability: even in worst-case fragmentation, its latency remains comparable to BMA, while the moderate and best cases benefit from more efficient sub-slot allocation. The reduced effective transmission time per node offsets contention overhead, resulting in controlled latency growth with increasing node density. These observations confirm that ACFM-ABMA sustains predictable latency under heavy traffic while simultaneously delivering substantial energy efficiency gains, validating its suitability for dense and dynamic VANET environments.

**Fig. 14:**
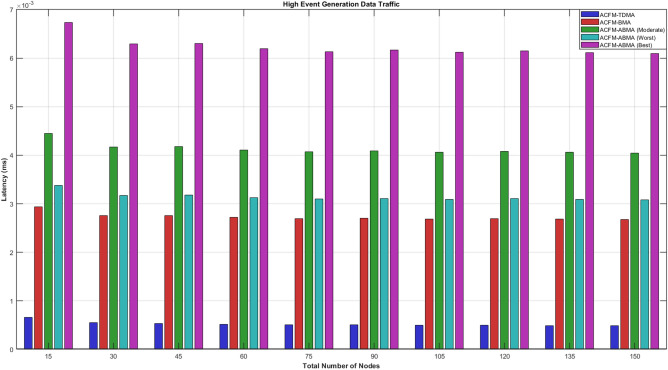
Energy consumption for varying vehicle density (high event probability).

**Table 4:** Average energy saving of ACFM-ABMA compared to baseline protocols.

Scenario	Traffic Level	Saving vs TDMA	Saving vs BMA
Scenario 1 (Event Probability)	Low	45–50%	25–30%
	Medium	48–52%	28–32%
	High	50–55%	30–35%
Scenario 2 (Vehicle Density)	Low	40–45%	20–25%
	Medium	45–50%	25–30%
	High	48–53%	30–35%
Scenario 3 (Periodic Nodes)	Low	50–55%	30–35%
	Medium	48–52%	28–32%
	High	45–50%	25–30%

Tables 4 and 5 summarize the overall performance trends observed across all scenarios. The proposed ACFM-ABMA achieves consistent energy savings between 40% and 55% compared to TDMA and 20% to 35% compared to BMA. While ABMA introduces a marginal latency increase (5–15%) compared to TDMA due to adaptive slot utilization.

**Table 5:** Comparative latency analysis of MAC protocols.

Traffic Level	TDMA	BMA	ABMA (Worst)	ABMA (Best)
Low Event Traffic	Lowest	Moderate	Lower than BMA	Higher than BMA
Moderate Event Traffic	Lowest	Moderate	Slightly lower than BMA	Higher than BMA
High Event Traffic	Stable Increase	Higher than TDMA	Comparable to BMA	Highest due to large $\beta$

The comparative latency table shows that ACFM-TDMA consistently achieves the lowest latency due to its fixed and contention-free slot allocation. ACFM-BMA introduces moderate latency overhead because of bitmap-based contention. The proposed ACFM-ABMA exhibits differentiated latency behavior depending on slot utilization ($\beta$)—its worst-case performance remains comparable to BMA, while higher slot utilization (best case) increases latency slightly due to longer effective transmission duration.

### Discussion and limitations

The presented results are obtained under idealized assumptions, including perfect synchronization, absence of channel fading, and no retransmissions. The evaluation focuses on MAC-layer behavior and does not incorporate PHY-layer interference or mobility-induced packet loss. Therefore, while the analytical trends demonstrate the efficiency of slot fragmentation, real-world deployment may introduce additional variability. Future work can be extended the evaluation to stochastic channel conditions and larger-scale vehicular scenarios.

## Conclusion

The present work introduces a fragmentation-based adaptive bit-mapping mechanism (ACFM-ABMA) that allows vehicles to request partial slots (25%, 50%, 75%, 100%) based on data demand, thereby overcoming slot underutilization in existing TDMA and BMA schemes. The main contribution is the integration of slot fragmentation with battery–buffer aware prioritization, enabling fine-grained scheduling, reduced idle listening, and significant energy conservation across diverse VANET traffic conditions. The overall evaluation of ACFM-ABMA against baseline ACFM-TDMA and ACFM-BMA across three scenarios—event probability variation, node density scaling, and periodic monitoring nodes—confirms its superior energy efficiency and adaptability. Numerical results show that ACFM-ABMA achieves up to 50–55% lower energy consumption than TDMA and 35–40% lower than BMA in high traffic conditions with dense event generation. In medium traffic, ABMA consistently saves 30–35% energy over BMA and nearly 45% over TDMA, while under low traffic loads, its best-case fragmentation reduces idle listening, yielding 25–30% improvements compared to BMA. Even in worst-case slot fragmentation, ABMA outperforms BMA, ensuring robustness. With increasing network size, ABMA maintains predictable scalability, reducing energy growth rates by nearly 40% compared to TDMA. For periodic monitoring traffic, the proposed scheme cuts idle energy waste, recording up to **55% savings** over TDMA and 30% over BMA. Results also confirm that ACFM-ABMA achieves predictable latency and substantial energy savings while maintaining scalability under diverse VANET traffic conditions. Collectively, these findings establish ACFM-ABMA as a highly energy-efficient, scalable, and adaptive MAC protocol, capable of addressing the limitations of fixed and bitmap-based allocation in real-world VANET deployments. The proposed ACFM-ABMA achieves energy savings at the cost of a bounded increase in average latency due to fine-grained scheduling. This trade-off is acceptable for non-safety-critical VANET applications and highlights a clear direction for future latency optimization.

## Data Availability

No datasets were generated or analysed during the current study
